# Personalized Consideration of Admission-Glucose Gap between Estimated Average and Initial Glucose Levels on Short-Term Stroke Outcome

**DOI:** 10.3390/jpm11020139

**Published:** 2021-02-18

**Authors:** Yerim Kim, Sang-Hwa Lee, Chulho Kim, Min Kyoung Kang, Byung-Woo Yoon, Tae Jung Kim, Jong Seok Bae, Ju-Hun Lee

**Affiliations:** 1 Department of Neurology, Kangdong Sacred Heart Hospital, Hallym University College of Medicine, Seoul 05355, Korea; brainyrk@hallym.ac.kr (Y.K.); jsbae69@gmail.com (J.S.B.); 2 Department of Neurology, Chuncheon Sacred Heart Hospital, Hallym University College of Medicine, Chuncheon 24253, Korea; neurolsh@hallym.or.kr (S.-H.L.); gumdol52@hallym.or.kr (C.K.); 3 Department of Neurology, Uijeongbu Eulji Medical Center, Eulji University College of Medicine, Uijeonbu-si 11759, Korea; eiri616@hanmail.net (M.K.K.); bwyoon@snu.ac.kr (B.-W.Y.); 4 Department of Neurology, Seoul National University College of Medicine, Seoul 03080, Korea; ttae35@gmail.com

**Keywords:** stroke, brain ischemia, hyperglycemia, glycated hemoglobin A, prognosis, glucose

## Abstract

Background: Poststroke hyperglycemia is associated with poor outcomes. Most prior studies used initial glucose as an indicator of poststroke hyperglycemia without considering glycemic control status at the time of stroke occurrence. We aimed to investigate the effect of an admission-glucose gap on short-term functional outcomes in acute ischemic stroke (AIS). Methods: We enrolled patients with AIS or transient ischemic attack who had been admitted within 7 days of symptom onset to three stroke centers from May 2016 to December 2019. The admission-glucose gap between estimated average glucose levels (eAG) and initial glucose level (eAG–initial glucose) was categorized into four groups. The short-term functional outcome was evaluated using the modified Rankin Scale (mRS) score at 3 months after stroke onset and was dichotomized. Results: Among 1332 included subjects, 548 (41.1%) had poor short-term functional outcomes. After adjusting for multiple variables, a severe negative glucose gap (eAG–initial glucose ≤ −50 mg/dL) was significantly associated with poor short-term functional outcome (OR, 1.573; 95% CI, 1.101–2.248). After dichotomizing glycemic control status, its significance was only maintained in the good glycemic control group (HbA1c < 6.5%) (OR, 1.914; 95% CI, 1.155–3.169). Conclusions: An elevated admission-glucose gap, in which the initial glucose level was much higher than the estimated glucose level was based on HbA1c, was associated with poor stroke prognosis. In addition to admission-glucose levels, glycemic control status at the time of stroke onset should be considered when predicting short-term stroke outcomes.

## 1. Introduction

The effect of variable glucose parameters on stroke outcomes is not yet clear. Previous studies reported that glycated hemoglobin (HbA1c), prestroke glycemic variability, and glucose dynamics after stroke were associated with stroke morbidity and mortality [1,2,3,4,5]. Hyperglycemia after stroke is common and occurs in 20% to 60% of patients with acute ischemic stroke (AIS) [6,7]. Although the association between poststroke hyperglycemia and outcomes remains controversial, poststroke hyperglycemia was shown to be associated with poor outcomes in several studies [3,8,9]. Although we cannot confirm the exact pathomechanism, according to the previous literature, hyperglycemia may affect stroke occurrence and outcome through modulating the pathway, including acidosis, free radical generation, inflammation, mitochondrial dysfunction, and other factors such as hypoxia inducible factor-1 (HIF-1) [10]. 

Poststroke hyperglycemia may provoke increased infarct volume [6], risk of hemorrhagic transformation [9,11], and recanalization failure after thrombolysis, thus leading to poor stroke outcomes [7,12]. Until now, most studies used random glucose, admission glucose, and fasting glucose levels as variables to assess poststroke hyperglycemia without consideration of glycemic control status at the time of stroke occurrence [3,8,13,14]. However, although there are various mechanisms of poststroke hyperglycemia development, the effect of hyperglycemia on stroke prognosis differs depending on glycemic control status at the time of stroke onset. Furthermore, it is difficult to clearly determine whether it is the effect of poststroke stress hyperglycemia or higher-than-usual glucose level at the time of stroke.

In the present study, we investigated the effect of the glucose gap, calculated on the basis of the difference between estimated average glucose level (eAG), calculated using HbA1c, and initial glucose level (eAG–initial glucose) on short-term functional outcome in AIS.

## 2. Materials and Methods

### 2.1. Study Population

**Figure 1:**
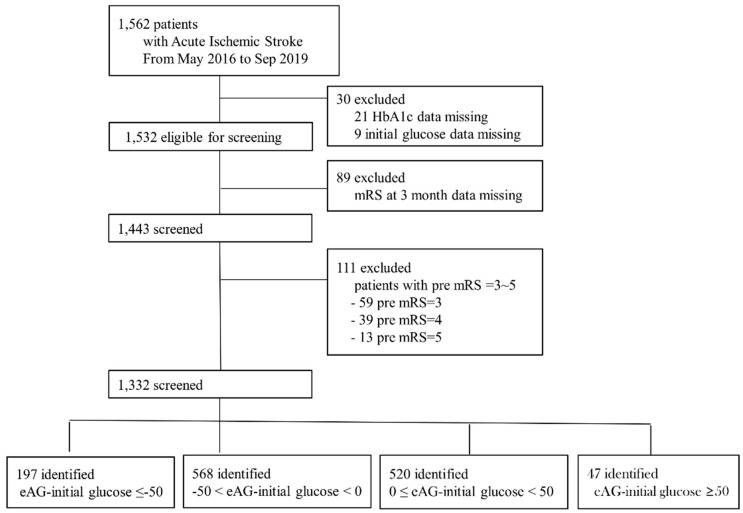
Flow diagram of study population.

We enrolled into our prospective stroke registry system patients with AIS or transient ischemic attack who had been admitted within 7 days of symptom onset to three stroke centers (Kangdong Sacred Heart Hospital, Hallym University College of Medicine; Chuncheon Sacred Heart Hospital, Hallym University College of Medicine; and Seoul National University Hospital) from May 2016 to December 2019. Among the 1562 patients, 30 subjects in whom the HbA1c (*n* = 21) or initial glucose (*n* = 9) levels were not evaluated were excluded. Additionally, 89 patients without a 3 month functional outcome capture and 111 patients with premodified Rankin Scale (pre-mRS) score ≥ 3 were excluded. As a result, we included a total of 1332 patients (Figure 1).

All patients received standard and optimal medical therapy during hospitalization. The institutional review boards of three centers (Kangdong Sacred Heart Hospital IRB no. 2020-02-006-001, Seoul National University Hospital IRB no. H-2002-023-1098, and Chuncheon Sacred Heart Hospital IRB no. 2017-89) approved the study protocol, and written informed consent was obtained from all participants or from their next of kin when the patient was unable to provide consent. 

### 2.2. Clinical Information

Baseline and demographic characteristics, including age and sex, risk factors (hypertension, diabetes, dyslipidemia, smoking history, and atrial fibrillation), stroke subtype according to Trial of Org 10,172 in Acute Stroke Treatment (TOAST) classification, imaging findings (computed tomography and/or magnetic resonance imaging), and laboratory data, were collected. Body weight and height were measured at admission. Body-mass index (BMI) was calculated as weight (kg) divided by height squared (m). 

Initial neurological severity (INS) was measured using the National Institute of Health Stroke Scale (NIHSS) score at admission. Short-term functional outcome was evaluated using the modified Rankin Scale (mRS) score at 3 months after stroke onset. Short-term functional outcomes were dichotomized (good outcome: 3 month mRS, 0–1; poor outcome: 3 month mRS, 2–6).

Initial glucose level was obtained upon admission. Glycated albumin (GA) and HbA1c levels were obtained after an overnight fast, and eAG was calculated using a validated formula [15]:

eAG (mg/dL) = 28.7 × A1c (mg/dL) − 46.7 or

eAG (mmol/L) = 1.59 × A1c (mmol/L) − 2.59 

The glucose gap between eAG and initial glucose level (eAG–initial glucose) was calculated, and patients were divided into four groups: mild-positive (0 ≤ eAG–initial glucose < 50 mg/dL), severe-positive (eAG–initial glucose ≥ 50 mg/dL), mild-negative (−50 < eAG–initial glucose < 0 mg/dL), and severe-negative (eAG–initial glucose ≤ −50 mg/dL; Figure 1) groups.

Glucose control status was divided into good (HbA1c < 6.5%) and poor (HbA1c ≥ 6.5%) glycemic control status.

### 2.3. Statistical Analysis

The distribution of demographic, clinical, laboratory, and stroke subtype data according to short-term functional outcomes was analyzed using a χ^2^ test, Student’s *t*-test, or Kruskal–Wallis test, as appropriate. The trend in the baseline data was also calculated using the χ^2^ test for trends in proportions. Associations between glucose gap and short-term functional outcomes were investigated using binary logistic-regression analysis. The mild-positive glucose gap group (0 ≤ eAG–initial glucose < 50 mg/dL) was used as a reference. 

Values for continuous variables are expressed as mean ± standard deviation (SD). Odds ratios (ORs) and 95% confidence intervals (CIs) are expressed for the results and probability values. A *p* value ≤ 0.05 was considered statistically significant. Analyses were performed using SPSS version 19.0 (SPSS Inc., Chicago, IL, USA).

### 2.4. Data-Availability Statement

Data cannot be shared because there are embargoes on datasets. Anonymized data will be shared by request from any qualified investigator.

## 3. Results

**Figure 2:**
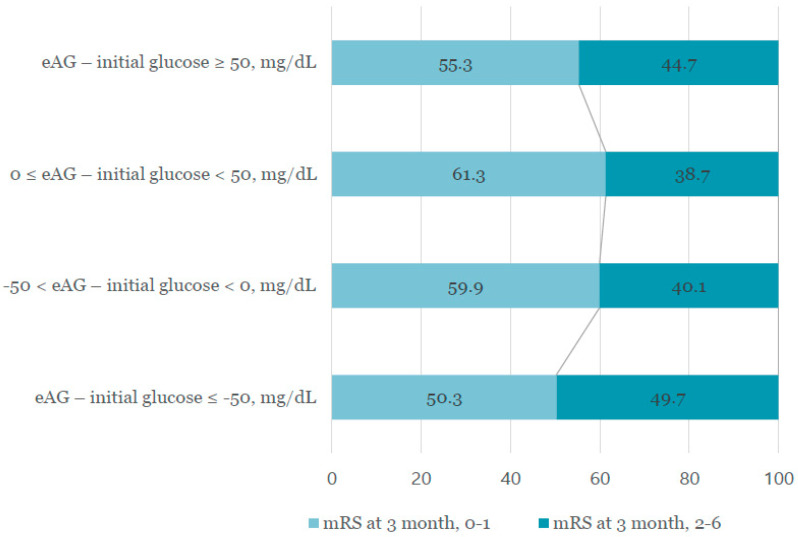
Distribution of levels of glucose gap groups after dichotomizing modified Rankin scale (mRS) at three months after stroke onset (mRS 0–1 versus mRS 2–6).

Between May 2016 and September 2019, 1332 patients were included in our study. Of these patients, 548 patients (41.1%) had poor short-term functional outcomes (Table 1). Those subjects were older, more likely to have a history of stroke, hypertension, diabetes, smoking history, or atrial fibrillation, and had cardioembolic or undetermined etiology. The categorized glucose gap group was distributed as follows: severe negative, 12.6% in good outcome versus 17.9% in poor outcome; mild negative, 43.4% versus 41.6%; mild positive, 40.7% versus 36.7%; and severe positive, 3.3% versus 3.8% (*p* = 0.044; Figure 2). Baseline and demographic characteristics (dichotomized according to short-term functional outcome) are shown in Table 1. Initial glucose level was 246.3 ± 80.2 mg/dL (severe-negative group), 140.4 ± 34.4 mg/dL (mild-negative group), 113.4 ± 30.4 mg/dL (mild-positive group), and 115.8 ± 36.0 mg/dL (severe-positive group). HbA1c was 7.02 ± 1.78% (severe-negative group), 5.89 ± 1.04% (mild-negative group), 6.17 ± 1.08% (mild-positive group), and 8.49 ± 1.58% (severe-positive group). There were no statistically significant differences in stroke mechanism or severity between groups (Table 2).

**Figure 3:**
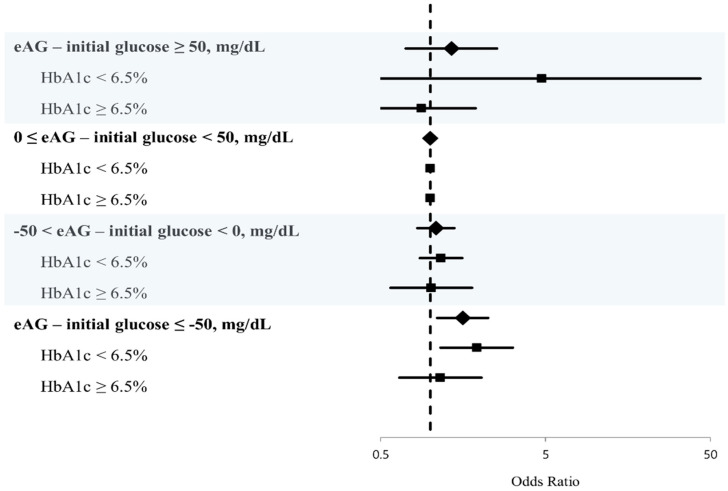
Logistic-regression plot of odds ratios and 95% confidence intervals. Effects of glucose gap on short-term stroke outcomes stratified by glycemic control status.

In binary logistic-regression analysis, we adjusted for multiple variables: age, sex, BMI, prior ischemic stroke, hypertension, diabetes, smoking, atrial fibrillation, hematocrit, blood urea nitrogen, high-sensitivity C-reactive protein, glucose gap between eAG and initial glucose, and initial stroke severity. Age, prior ischemic stroke, atrial fibrillation, initial stroke severity, and glucose gap were associated with poor functional outcome (3 month mRS, 0–1). Among glucose gap groups, the severe-negative group (eAG–initial glucose ≤ −50, mg/dL) was significantly associated with poor short-term functional outcome (OR, 1.573; 95% CI, 1.101–2.248; Table 3 and Figure 3). Additionally, we conducted binary logistic-regression analysis only in the female group and had similar results (supplementary Table S1).

After categorization by glycemic control status (HbA1c < 6.5% versus HbA1c ≥ 6.5%), the severe-negative group (eAG–initial glucose ≤ −50 mg/dL) was significantly associated with poor functional outcome (OR, 1.914; 95% CI, 1.155–3.169) in the good glycemic control status group (HbA1c < 6.5%), whereas the association was not significant in the poor glycemic control status group (Table 4 and Figure 3). 

## 4. Discussion

As far as we are aware, this study is the first to investigate the admission glucose gap between eAG and initial glucose level in association with stroke outcome considering glycemic control status at the time of stroke onset. We identified that patients with a severe negative glucose gap (eAG–initial glucose ≤ −50 mg/dL) were significantly associated with poor short-term functional outcome. Its significance was only maintained in the good glycemic control group (HbA1c < 6.5%). This finding suggests that initial glucose level might have limited prognostic importance as a predictor of short-term stroke outcome without reflecting glycemic control status. 

Several clinical studies demonstrated an association between admission hyperglycemia and poor stroke outcome [1,2,3,6,8]. It was proposed that elevated glucose causes infarct evolution through anaerobic glycolysis, leading to lactic acidosis, reperfusion-induced superoxide production [16], increased matrix-metalloproteinase-9 expression, and provoking blood–brain barrier damage [17,18]. Most previous studies concentrated on initial glucose level without considering the estimated glucose level at the time of stroke occurrence [13,14]. However, the initial glucose level should be considered in light of glycemic control status at the time of stroke onset. The effect of hyperglycemia at admission between patients with good and poor glycemic control status on stroke outcome might be different. Supporting our suggestion, in the second European Cooperative Acute Stroke Study (ECASS-II), hyperglycemia at admission was not related to any stroke outcomes, including 7 day neurological improvement, 30 day Barthel Index, 90 day mRS, and 90 day death [3]. In this regard, we demonstrated that a severe negative glucose gap between eAG and initial glucose was a significant predictor for poor short-term functional outcome. 

Although initial glucose level has an effect on stroke prognosis, it is difficult to clearly determine whether it is the effect of poststroke stress hyperglycemia or the effect of higher-than-usual glucose level at the time of stroke. Interestingly, in this study, the initial glucose level of the severe-negative group was the highest, but there was no statistically significant difference in stroke severity or mechanism between groups (Table 2). Therefore, considering that stroke severity is related to stress hyperglycemia, initial glucose status at the time of stroke rather than poststroke stress hyperglycemia may be an important predictor of short-term stroke prognosis. 

Furthermore, the group with poor glycemic control (HbA1c ≥ 6.5%) had poor prognosis, as expected, whereas the glucose gap affected short-term outcomes, particularly in the group without diabetes or with good glycemic control (HbA1c < 6.5%). Similar to our result, although the association between admission glucose level and stroke outcome was inconsistent, a systematic review of 31 articles demonstrated that the relationship between admission hyperglycemia and mortality was stronger in nondiabetic stroke patients than it was in diabetic patients (RR, 3.07 versus 1.3) [7]. There are some explanations for this finding. First, because patients with uncontrolled glucose have a higher baseline glucose level, threshold values affecting short-term functional outcomes might be different between the two groups. In our study, the mean value of the glucose gap between eAG and initial glucose was −10.03 ± 32.75 mg/dL in the good glycemic control group (HbA1c < 6.5%) and −17.58 ± 68.76 mg/dL in the poor glycemic control group (HbA1c ≥ 6.5%) (*p* = 0.034). Second, chronic hyperglycemic exposure may provoke cerebrovascular remodeling [19]. Glycemic control status seems to have different effects on microvascular and macrovascular complications [20]. Third, glucose-lowering drugs may reduce the toxic metabolic effect of hyperglycemia upon admission. 

It remains undetermined why HbA1c is not associated with short-term functional outcomes in our study. Reported associations between HbA1c levels and short-term stroke outcomes were inconsistent. In a retrospective study of 317 diabetic patients with coronary syndrome, HbA1c before admission was not associated with inhospital mortality or 6 month major adverse cardiovascular events [21]. In 608 patients with myocardial infarction, HbA1c was not an independent predictor of short-term outcomes, including 7 and 30 day mortality [1]. However, in a total of 534 patients with AIS undergoing mechanical thrombectomy, HbA1c was an independent predictor of poor short-term functional outcome [2]. Although the exact pathomechanism is unclear because HbA1c is a good indicator of glycemic control over 2 or 3 months, HbA1c seems to be a better marker of long-term outcomes and mortality than of short-term outcomes.

The strength of this study is that it was the first to investigate the effect of a glucose gap on stroke prognosis by considering glycemic control status at the time of stroke onset. Another advantage was the large population of the multicenter studies. Despite these strengths, there were a few limitations to this study. First, it was an observational retrospective study. Second, missing data may influence the interpretation of the results. However, we included a relatively large population from three tertiary hospitals to reduce selection bias. Third, poststroke hyperglycemia may be a secondary stress response after stroke. We cannot distinguish stress hyperglycemia from hyperglycemic status at the time of stroke onset. However, accumulating evidence suggests that poststroke hyperglycemia is not merely an epiphenomenon; rather, it has a critical pathologic role in stroke evolution. One study based on magnetic resonance imaging reported on the correlation of acute hyperglycemia with perfusion–diffusion mismatch, greater infarct size, and poor functional outcomes [22]. Another study reported that increased serum glucose level (>8.9 mmol/L) triggers antifibrinolytic cascades, contributing to hypercoagulable status [23]. Accordingly, in our study, there were no statistically significant differences in stroke mechanism or severity between groups that could cause stress hyperglycemia. Fourth, since this was a clinical retrospective study, we did not measure some biomarkers to better understand how the glucose gap affected stroke outcome. Recent studies demonstrated that the multifaceted role of mitochondria, free radical generation, and inflammatory cytokines can affect ischemic brain injury [10,24,25]. In order to advance our research, it is better to consider such experimental parameters in future research. Lastly, although we obtained all initial glucose levels on the day of admission, day of admission and stroke onset were different for each patient. However, since we enrolled patients with AIS who had been admitted within 7 days of symptom onset, duration differences were limited. 

## 5. Conclusions

In conclusion, an elevated initial glucose gap, of which the initial glucose level is much higher than the estimated glucose level is based on HbA1c, was associated with poor stroke prognosis. In addition to glucose level at admission, glycemic control status at the time of stroke onset should be considered in the prediction of short-term stroke outcomes. 

## Figures and Tables

**Table 1:** Baseline characteristics according to initial stroke severity.

	Good Outcome (3 Month mRS 0–1)	Poor Outcome (3 Month mRS 2–6)	*p* Value
No. (%)	784 (58.9)	548 (41.1)	
Age, years	66 ± 13	71 ± 12	<0.001
Male sex, %	518 (66.1)	314 (57.3)	0.001
Body-mass index, kg/m^2^	24.2 ± 3.3	23.8 ± 3.6	0.038
Cardiovascular risk factor			
Prior ischemic stroke	131 (16.7)	121 (22.1)	0.014
Hypertension	459 (58.5)	352 (64.2)	0.036
Diabetes	245 (31.3)	210 (38.3)	0.007
Dyslipidemia	155 (19.8)	100 (18.2)	0.487
Smoking	228 (29.1)	103 (18.8)	<0.001
Atrial fibrillation	96 (12.2)	124 (22.6)	<0.001
Laboratory			
White-blood-cell count	7778 ± 3482	7900 ± 2979	0.507
Hematocrit, g/dL	41.1 ± 5.5	40.0 ± 6.4	0.001
Fasting blood sugar, mg/dL	128.5 ± 61.8	144.1 ± 88.2	<0.001
HbA1c, %	6.19 ± 1.23	6.36 ± 1.48	0.027
Estimated average glucose (eAG), mg/dL	130.8 ± 35.2	135.7 ± 42.4	0.027
eAG–initial glucose, mg/dL	−10.26 ± 43.40	−14.11 ± 48.89	0.139
eAG–initial glucose–four groups			0.044 *
eAG–initial glucose ≤ −50, mg/dL	99 (12.6)	98 (17.9)	
−50 < eAG–initial glucose < 0, mg/dL	340 (43.4)	228 (41.6)	
0 ≤ eAG–initial glucose < 50, mg/dL	319 (40.7)	201 (36.7)	
eAG–initial glucose ≥ 50, mg/dL	26 (3.3)	21 (3.8)	
Initial glucose, mg/dL	141.1 ± 58.5	149.8 ± 65.7	0.013
Glycated albumin, %	16.0 ± 4.4	17.2 ± 4.8	<0.001
High-sensitivity C-reactive protein, mg/L	6.49 ± 23.03	10.33 ± 26.60	0.008
Total cholesterol, mg/dL	171.1 ± 43.3	166.8 ± 42.7	0.026
Triglyceride, mg/dL	144.2 ± 106.2	127.0 ± 97.1	0.002
Low-density lipoprotein, mg/dL	104.4 ± 35.4	102.3 ± 51.6	0.395
Blood urea nitrogen, mg/dL	16.8 ± 8.2	18.6 ± 11.2	0.001
Creatinine, mg/dL	0.99 ± 0.80	1.04 ± 0.84	0.298
Systolic blood pressure, mmHg	152 ± 29	152 ± 29	0.597
Diastolic blood pressure, mmHg	86 ± 15	85 ± 16	0.91
Mechanism			<0.001 *
Large artery atherosclerosis	266 (34.5)	195 (35.9)	
Small vessel occlusion	240 (31.0)	109 (20.1)	
Cardioembolic	114 (14.8)	115 (21.2)	
Other determined	33 (4.3)	22 (4.1)	
Undetermined	119 (15.4)	102 (18.8)	
Previous antiplatelet user, n (%)	208 (26.5)	158 (28.8)	0.312
Previous anticoagulant user, n (%)	25 (3.2)	33 (6.0)	0.043
Initial stroke severity, median (IQR)	2 (1,5)	4 (2,7)	<0.001

Abbreviations: IQR, interquartile range. No. (%) or mean ± standard deviation (SD). *p* values calculated using χ^2^ test for trends in proportions. * Linear by linear association was used for analysis.

**Table 2:** Baseline characteristics grouped by weight change.

	eAG–Initial Glucose ≤ −50, mg/dL	−50 < eAG–Initial Glucose < 0, mg/dL	0 ≤ eAG–Initial Glucose < 50, mg/dL	eAG–Initial Glucose ≥ 50, mg/dL	*p* Value
No. (%)	197 (14.8)	568 (42.6)	520 (39.0)	47 (3.5)	
Age, years	69.5 ± 11.6	68.1 ± 13.7	68.0 ± 12.5	68.7 ± 12.8	0.712
Male sex, %	122 (61.9)	364 (64.1)	318 (61.2)	28 (59.6)	0.751
Body mass index, kg/m^2^	24.40 ± 3.60	23.83±3.43	23.96 ± 3.37	24.62 ± 3.88	0.248
Cardiovascular risk factor					
Prior ischemic stroke	37 (18.8)	99 (17.4)	106 (20.4)	10 (21.3)	0.632
Hypertension	121 (61.4)	346 (60.9)	308 (59.2)	36 (76.6)	0.139
Diabetes	116 (58.9)	146 (25.7)	159 (30.6)	34 (72.3)	<0.001
Dyslipidemia	39 (19.8)	95 (16.7)	109 (21.0)	12 (25.5)	0.208
Smoking	47 (23.9)	140 (24.6)	132 (25.4)	12 (25.5)	0.976
Atrial fibrillation	36 (18.3)	81 (14.3)	93 (17.9)	10 (21.3)	0.260
Laboratory					
White blood cell count	8052 ± 2981	7812 ± 2554	7736 ± 4008	8099 ± 3484	0.258
Hematocrit, g/dL	39.8 ± 6.1	40.8 ± 5.9	40.7 ± 5.1	42.1 ± 10.3	0.321
Fasting blood sugar, mg/dL	193.1 ± 108.6	129.9 ± 56.6	119.2 ± 66.7	129.6 ± 49.0	<0.001
HbA1c, %	7.02 ± 1.78	5.89 ± 1.04	6.17 ± 1.08	8.49 ± 1.58	<0.001
HbA1c ≥ 6.5%	101 (51.3)	109 (19.2)	130 (25.0)	43 (91.5)	<0.001
Initial glucose, mg/dL	246.3 ± 80.2	140.4 ± 34.4	113.4 ± 30.4	115.8 ± 36.0	<0.001
Glycated albumin, %	19.3 ± 6.1	15.6 ± 3.6	15.9 ± 3.8	22.6 ± 6.8	<0.001
Glycated albumin > 15.5%	114 (65.9)	201 (39.7)	189 (43.9)	36 (85.7)	<0.001
High-sensitivity C-reactive protein, mg/L	11.69 ± 33.57	8.52 ± 26.83	6.21 ± 17.36	8.67 ± 22.49	0.105
Total cholesterol, mg/dL	168.8 ± 44.3	171.9 ± 44.3	166.3 ± 41.3	161.3 ± 41.8	0.153
Triglyceride, mg/dL	155.7 ± 111.6	137.5 ± 106.1	129.2 ± 96.1	141.8 ± 86.2	0.010
Low-density lipoprotein, mg/dL	100.7 ± 34.7	105.2 ± 35.5	103.0 ± 52.2	101.7 ± 39.2	0.357
Blood urea nitrate, mg/dL	19.2 ± 11.1	17.2±9.2	17.0 ± 8.7	21.3 ± 14.4	0.006
Creatinine, mg/dL	1.07 ± 0.87	0.99 ± 0.82	0.99 ± 0.71	1.28 ± 1.45	0.100
Systolic blood pressure, mmHg	151 ± 31	152 ± 30	152 ± 26	146 ± 29	0.230
Diastolic blood pressure, mmHg	83 ± 15	86 ± 16	86 ± 14	80 ± 15	0.006
Mechanism					0.635 *
Large artery atherosclerosis	71 (36.0)	211 (37.5)	159 (31.2)	20 (42.6)	
Small vessel occlusion	42 (21.3)	149 (26.5)	149 (29.2)	9 (19.1)	
Cardioembolic	34 (17.3)	87 (15.5)	98 (19.3)	10 (21.3)	
Other determined	11 (5.6)	25 (4.4)	18 (3.5)	1 (2.1)	
Undetermined	39 (19.8)	90 (16.0)	85 (16.7)	7 (14.9)	
Previous antiplatelet user, n (%)	53 (26.9)	133 (23.4)	165 (31.7)	15 (31.9)	0.071
Previous anticoagulant user, n (%)	11 (5.6)	20 (3.5)	25 (4.8)	2 (4.3)	0.355
Initial stroke severity, median (IQR)	3 (1,6)	3 (1,6)	3 (1,6)	3 (2,5)	0.883
3 month mRS 2–6	98 (49.7)	228 (40.1)	201 (38.7)	21 (44.7)	0.050

Abbreviations: IQR, interquartile range. No. (%) or mean ± SD. *p* values calculated using χ^2^ for trends in proportions. * Linear by linear association was used for analysis.

**Table 3:** Binary logistic-regression analysis for poor short-term functional outcomes, mRS 2–6, 3 months after stroke (compared to mRS 0–1, 3 months after stroke).

	Odds Ratio (95% Cl)	*p* Value
Male sex	0.854 (0.653–1.117)	0.248
Age, years	1.023 (1.012–1.034)	<0.001
Body mass index, kg/m^2^	0.985 (0.951–1.020)	0.392
Conventional risk factors		
Prior ischemic stroke	1.384 (1.030–1.859)	0.031
Hypertension	0.974 (0.753–1.259)	0.840
Dyslipidemia	0.910 (0.672–1.231)	0.541
Smoking	0.846 (0.621–1.153)	0.289
Atrial fibrillation	1.534 (1.120–2.101)	0.008
Hematocrit, g/dL	1.003 (0.981–1.026)	0.794
Blood urea nitrogen, mg/dL	1.010 (0.997–1.023)	0.116
High-sensitivity C-reactive protein, mg/L	1.004 (0.999–1.009)	0.136
eAG–initial glucose, four groups		
eAG–initial glucose ≤ −50 mg/dL	1.573 (1.101–2.248)	0.013
−50 < eAG–initial glucose < 0 mg/dL	1.082 (0.836–1.400)	0.551
0 ≤ eAG–initial glucose < 50 mg/dL	reference	
eAG–initial glucose ≥ 50 mg/dL	1.344 (0.711–2.540)	0.363
Initial stroke severity	1.039 (1.016–1.062)	0.001

Abbreviation: eAG, estimated average glucose. Adjusted for age, sex, body mass index, previous stroke history, hypertension, dyslipidemia, smoking, atrial fibrillation, hematocrit, blood urea nitrogen, high-sensitivity C-reactive protein, glucose gap, and initial stroke severity.

**Table 4:** Binary logistic-regression analysis for poor short-term functional outcomes after categorization by glycemic control status (HbA1c < 6.5% versus HbA1c ≥ 6.5%).

	Odds Ratio (95% Cl)	*p* Value
HbA1c < 6.5%		
eAG–initial glucose, four groups		
eAG–initial glucose ≤ −50, mg/dL	1.914 (1.155–3.169)	0.012
−50 < eAG–initial glucose < 0, mg/dL	1.161 (0.864–1.562)	0.322
0 ≤ eAG–initial glucose < 50, mg/dL	Reference	
eAG–initial glucose ≥ 50, mg/dL	4.738 (0.464–43.384)	0.189
**HbA1c ≥ 6.5%**		
eAG–initial glucose, four groups		
eAG–initial glucose ≤ −50, mg/dL	1.152 (0.650–2.042)	0.628
−50 < eAG–initial glucose < 0, mg/dL	1.013 (0.574–1.788)	0.965
0 ≤ eAG–initial glucose < 50, mg/dL	Reference	
eAG–initial glucose ≥ 50, mg/dL	0.883 (0.415–1.881)	0.747

Abbreviations: CI, confidence interval; eAG, estimated average glucose. Adjusted for age, sex, body-mass index, stroke history, hypertension, dyslipidemia, smoking, atrial fibrillation, hematocrit, blood urea nitrogen, high sensitivity C-reactive protein, glucose gap, and initial stroke severity.

## Data Availability

All data generated or analyzed during this study are included in this published article. Anonymized data will be shared by reasonable request from any qualified investigator.

## Supplementary Materials

The following are available online at https://www.mdpi.com/2075-4426/11/2/139/s1, Supplementary Table S1: Binary logistic regression analysis for poor short-term functional outcome, mRS 2–6 three months after stroke (compared to mRS 0–1 three months after stroke) in the female group.

## Abbreviations

AIS	acute ischemic stroke
eAG	estimated average glucose levels
mRS	modified Rankin Scale
OR	odds ratio
HbA1c	glycated hemoglobin
TOAST	Trial of Org 10172 in Acute Stroke Treatment
BMI	body-mass index
INS	initial neurological severity
NIHSS	National Institute of Health Stroke Scale (NIHSS) score
GA	glycated albumin
SD	standard deviation
CIs	confidence intervals
ECASS-II	second European Cooperative Acute Stroke Study
