# Supplementary material for: Personalized Consideration of Admission-Glucose Gap between Estimated Average and Initial Glucose Levels on Short-Term Stroke Outcome
Source: J Pers Med. 2021 Feb 18;11(2):139. doi: 10.3390/jpm11020139 (PMC7922921; doi:10.3390/jpm11020139)
Supplement: Supplementary file 1 [file jpm-11-00139-s001.pdf]

Supplementary material

Personalized Consideration of the Admission Glucose Gap between Estimated Average and Initial Glucose Levels on Short-term Stroke Outcome

Yerim Kim,[1] Sang-Hwa Lee,[2] Chulho Kim,[2] Min Kyung Kang,[3] Byung-Woo Yoon,[3] Tae Jung Kim,[4] Jong Seok Bae,[1] Ju-Hun Lee[1,*]

[1]Department of Neurology, Kangdong Sacred Heart Hospital, Hallym University College of Medicine, Seoul 05355, Korea; brainyrk@hallym.ac.kr(Y.K.); leejuhun@kdh.or.kr (J.-H.L.); jsbae69@gmail.com (J.S.B.)
[2]Department of Neurology, Chuncheon Sacred Heart Hospital, Hallym University College of Medicine, Chuncheon 24253, Korea; neurolsh@hallym.or.kr(S.-H.L.) and gumdol52@hallym.or.kr (C.K.)
[3]Department of Neurology, Uijeongbu Eulji Medical Center, Eulji University College of Medicine, Uijeonbu-si 11759, Korea; eiri616@hanmail.net (M.K.K.) and bwyoon@snu.ac.kr (B.-W.Y.)
[4]Department of Neurology, Seoul National University College of Medicine, Seoul 03080, Korea; ttae35@gmail.com (T.J.K.)

Supplementary table. Binary logistic regression analysis for poor short-term functional outcome, mRS 2–6 three months after stroke (compared to mRS 0–1 three months after stroke) in the female group

	Odds ratio (95% Cl)	p-value
Age, years	1.044 (1.024–1.064)	**<0.001**
Body mass index, kg/m^2	0.992 (0.939–1.047)	0.763
Conventional risk factors		
Prior ischemic stroke	1.268 (0.746–2.156)	0.380
Hypertension	0.940 (0.589–1.501)	0.796
Dyslipidemia	0.967 (0.586–1.595)	0.896
Smoking	2.125 (0.768–5.880)	0.147
Atrial fibrillation	1.913 (1.156–3.167)	**0.012**
Hematocrit, g/dL	1.005 (0.971–1.041)	0.758
Blood urea nitrogen, mg/dL	0.994 (0.972–1.017)	0.611
High-sensitivity C-reactive protein, mg/L	1.010 (1.001–1.019)	0.026
eAG - initial glucose, four groups		
eAG - initial glucose ≤-50 mg/dL	2.933 (1.566–5.492)	**0.001**
-50< eAG - initial glucose <0 mg/dL	1.119 (0.727–1.722)	0.610
0≤ eAG - initial glucose <50 mg/dL	reference	
eAG - initial glucose ≥50 mg/dL	1.958 (0.677–5.662)	0.215
Initial stroke severity	1.057 (1.018–1.097)	**0.001**

Abbreviation: eAG, estimated average glucose

Adjusted for age, sex, body mass index, previous stroke history, hypertension, dyslipidemia, smoking, atrial fibrillation, hematocrit, blood urea nitrogen, high-sensitivity C-reactive protein, glucose gap, and initial stroke severity
